# Whole Exome Sequencing in 16p13.11 Microdeletion Patients Reveals New Variants Through Deductive and Systems Medicine Approaches

**DOI:** 10.3389/fgene.2022.798607

**Published:** 2022-03-15

**Authors:** Paola Granata, Dario Cocciadiferro, Alessandra Zito, Chiara Pessina, Alessandro Bassani, Fabio Zambonin, Antonio Novelli, Mauro Fasano, Rosario Casalone

**Affiliations:** ^1^ Cytogenetics and Medical Genetics Unit, Department of Services, ASST dei Sette Laghi, Varese, Italy; ^2^ Laboratory of Medical Genetics, Translational Cytogenomics Research Unit, Ospedale Pediatrico Bambino Gesù, Roma, Italy; ^3^ Department of Medicine and Surgery, University of Insubria, Varese, Italy; ^4^ Child Neuropsychiatry Unit, Department of Maternal and Child Health, ASST dei Sette Laghi, Varese, Italy; ^5^ Department of Science and High Technology and Center of Bioinformatics, University of Insubria, Busto Arsizio, Italy

**Keywords:** 16p13.11 microdeletion, copy number variants (CNVs), whole exome sequencing (WES), neurodevelopmental disorders, protein-protein interactions (PPIs)

## Abstract

The 16p13.11 microdeletion, whose prevalence in the general population is about 0.04%, is known in literature as a predisposition factor to neurodevelopmental disorders, being found in about 0.13% of patients with schizophrenia, in 0.5–0.6% of patient with epilepsy, cognitive impairment, autism spectrum disorder (ASD) and aggressiveness. The goal of this study was to identify a specific gene set pattern unique for the affected patients in comparison with other familial components. Due to the incomplete penetrance of this copy number variant (CNV), we studied by whole exome sequencing (WES), with particular regard of 850 SFARI genes, three families with an affected member carrier of inherited 16p13.11 and 16p13.11p12.3 microdeletion and one family with an affected member with a *de novo* 16p13.11 microdeletion. By combining a deductive approach together with personalized network models, we identified gene signatures potentially capable of explaining the clinical phenotype. Candidate variants in genes of interest were identified as possibly involved in determining the neurological phenotype of the four patients, such as compound heterozygosity in *CECR2*, variants in *MTOR* and *RICTOR* genes, compound heterozygous single nucleotide variants in the *LRRK2* gene. Moreover, genes present in the microdeletion region were partially present as central nodes, with a focus on *NDE1*. No additional pathogenetic or uncertain CNVs were found in all four patients. No significant variants were detected in genes included in the microdeletion in patients 1, 2 and 3, excluding the finding of unmasked recessive variants. In conclusion, WES is a fundamental tool in the genetic investigation of patients having a predisposing variant, which is not sufficient to define the clinical phenotype. Moreover, the analysis of WES data using Systems medicine tools, such as personalized network models, led to the prioritization of genes on a high throughput scale and to discover variants in genes that were not prioritized at first.

## Introduction

The microdeletion 16p13.11 (chr16:15.48-16.32 Mb, GRCh37/hg19) is a well known genomic rearrangement previously reported as predisposing to neurodevelopmental disorders (Ullmann et al., 2007; Hannes et al., 2009; Law et al., 2009; de Kovel et al., 2010; Heinzen et al., 2010; Mefford et al., 2010; Alkuraya et al., 2011; Balasubramanian et al., 2011; Ingason et al., 2011; Nagamani et al., 2011; Paciorkowski et al., 2013; Tropeano et al., 2013; Tropeano et al., 2014). Genes mapped in the deleted region include *NDE1*, the strongest candidate gene for neurodevelopmental phenotype and microcephaly, expressed in brain, which protein plays an essential role in microtubule organization, mitosis and neuronal migration. The region also includes *NTAN1*, involved in protein degradation and related to altered behavior, and *MYH11*, coding for the major contractile protein in smooth muscle cells [GeneCards (Stelzer et al., 2016)].

The microdeletion is found in about 0.13% of patients with schizophrenia (Ingason et al., 2011), in 0.5–0.6% of patients with epilepsy (de Kovel et al., 2010; Heinzen et al., 2010; Ingason et al., 2011) and it has been associated with a wide spectrum of multiple congenital anomalies such as facial dysmorphisms and different cognitive impairment, autism spectrum disorder (ASD), and aggressiveness (www.rarechromo.org). However, since the prevalence of the variation in the general population is about 0.04% (1:2,300–2,500 individuals) (Paciorkowski et al., 2013; Tropeano et al., 2014) and the variation is frequently present in unaffected parents and relatives of affected subjects, the pathological significance of 16p13.11 microdeletion remains uncertain. The association between abnormal phenotype and 16p13.11 microdeletion could be a mere coincidence. From a meta analysis by Rosenfeld and coworkers, the del16p13.11 frequency in postnatal array comparative genomic hybridization (aCGH) cases is 0.15%, while the frequency in controls is 0.05%. The *de novo* occurence in cases has a frequency of 21.7%, with a penetrance estimate of 13.1% (95% CI) (Rosenfeld et al., 2013). Indeed, the incomplete penetrance frequently observed in del16p13.11 families may be due to variable phenotypic expressivity, phenomenon of unmasking of a recessive variant, variants in genes outside the deletion, different extension of the microdeletion or a combination of all these events.

In order to identify the presence and significance of a possible second hit mutational event or other pathological variants in genes outside the deleted region, we performed whole exome sequencing (WES) in four del16p13.11 patients and their unaffected parents and siblings with the same microdeletion. The WES results were analyzed with two distinct approaches, in parallel. First, the classical deductive approach led to the identification of potentially relevant variants. Next, the inductive approach that characterizes Systems medicine allowed us to discover new variants in genes that were not prioritized at first. Altogether, results contributed to better define the clinical relevance of del16p13.11 and to shed light on the mechanisms of incomplete penetrance and of phenotypic heterogeneity.

## Materials and Methods

### Patients

Four patients with 16p13.11 microdeletion were referred to the Medical Genetics Unit of “ASST dei Sette Laghi” Hospital (Varese, Italy) for genetic investigation and counselling, with medical indication of developmental disorders, learning delay, intellectual disability, with or without congenital dysmorphic features. This study was based on results obtained for diagnostic purpose. A written informed consent was provided by the parents and relatives of the patients included in this study to perform aCGH and WES and to use the data for research.

#### Patient 1

Male patient, born at term without neonatal or prenatal distress, with weight = 3.70 kg, length = 52 cm, CC = 35 cm and APGAR = 9/10. He showed normal development, normal achievement of development milestones and no motor abnormalities; at the age of 8 he showed growth deficiency and dysmorphisms including low-set auricles, slow down eyelid rim, ogival palate with dental malocclusion, lower thick and down slated lip. The patient at this age developed difficulties in fine motor skills, showed low I.Q. (WISC-IV I.Q. 86), impulsiveness, spatial visualization difficulties, reading and understanding difficulties, dyscalculia, dysgraphia, spelling skills <5^th^ percentile, mathematical difficulties and memory deficit, TCM rapidity <10^th^ percentile, minimal atrioventricular valve insufficiency. At the age of 12, the patient showed hypotonia, persistent dysmorphism, epicanthus, scoliosis, winged shoulder blades, unilateral cryptorchidism, thinness, anxiety. No epilepsy was detected. The karyotype was 46,XY.

#### Patient 2

Male patient, born to term with caesarean section after normal pregnancy, with weight = 3.32 kg, length = 49 cm and CC = 34 cm. At the age of 5 he showed medium degree intellectual deficit (WISC-IV I.Q. 40), hypotonia, developmental and severe language delay, aggressivity and obsessive crises. He showed facial dysmorphisms (such as carp mouth), obesity, hyperphagia, but no hypogenitalism. Audiometric tests, MRI and EEG were normal. No epilepsy was detected. The karyotype was 46,XY and the methylation test for Prader-Willi/Angelman syndrome showed normal biparental methylation pattern.

#### Patient 3

Female patient, born at the 38^th^ week of gestation with caesarean section, with weight = 3.42 kg, length = 49 cm, CC = 34 cm and no malformations. At the age of 8 she presented dysorthography (ICD-10 F81.1) and dysgraphia in evolution (ICD-10 F81.8) with associated expressive language disorder (ICD-10 F80.1); the intellectual level was at its lower limit (WISC-IV I.Q. 81). Psychomotor development was normal. The patient showed normal height (80^th^ percentile) and no dysmorphisms. No epilepsy was detected (normal EEG). The karyotype was 46,XX.

#### Patient 4

Male patient, born at the 42^nd^ week of gestation with weight = 3.66 kg, length = 55, CC = 36, APGAR = 9/10. He showed normal psychomotor development and no dysmorphic features. The intellectual level was low (WISC-IV I.Q. 80). At the age of 5 he revealed severe global developmental and language delay and stereotypies. The diagnosis of non-syndromic ASD was assessed. No epilepsy was detected (normal EEG). The karyotype was 46,XY.

### Array CGH

Array CGH was performed for all the components of the four families after DNA extraction (QIAmp DNA blood Maxi Kit, Qiagen, Hilden, Germany) from patients and relatives peripheral blood cells. CytoSure ISCA V2 4x180K platform with a backbone resolution of 1 probe/25 Kb and 1 probe/19 Kb in critical regions, human genome reference GRCh37/hg19 and sex matched normal human DNA pool (Kreatech, Amsterdam, Netherlands) as control were used. InnoScan 710 Microarray Scanner (Innopsys, Carbonne, France) and Mapix (Innopsys, Carbonne, France) were used to detect and analyze fluorescence levels, respectively. Results were interpreted using Cytosure Interpret Software (Oxford Gene Technology, Begbroke, United Kingdom). QC metrics: SD < 1.0 and DLR spread <0.3 were required. The significance of CNVs (copy number variants) was evaluated according to American College of Medical Genetics (ACMG) Joint Consensus (Riggs et al., 2012).

### Whole Exome Sequencing

WES was performed on all the members of the four families using the Twist Human Core Exome Kit (Twist Bioscience, San Francisco, United States) according to the manufacturer’s protocol and sequenced with the Illumina NovaSeq 6000 platform. The BaseSpace pipeline (Illumina, San Diego, United States) and the TGex software (LifeMap Sciences, Alameda, United States) were used for the variant calling and annotation, respectively. Sequencing data were aligned to the GRCh37/hg19 human reference genome. Variants with a coverage lower than 10×, genotype quality (GQ) < 15, and gnomAD minor allele frequency (MAF) > 5% were excluded. WES results were interpreted according to ACMG guidelines 2015 (Richards et al., 2015).

### Analyzed Genes

WES was applied to investigate: 850 neurodevelopmental genes from SFARI database, genes showing pathogenetic or likely pathogenetic variants following the American College of Medical Genetics and Genomics (ACMG) classification (Richards et al., 2015), genes included in the 16p13.11 and 16p1.11p12.3 microdeletions (*NOMO1, PDXDC1, NTAN1, RRN3, MPV17L, MARF1, NDE1, MYH11, CEP20, ABCC1, ABCC6, NOMO3, XYLT1*) and genes involved with *NDE1* in cargo transport along the axon (i.e., *PAFAH1B1, DCTN1, DCTN2, DCTN3, DCTN4, DCTN5, DCTN6, PIK3C3,* and *PPP1R10*) (Kuijpers et al., 2016; Monda and Cheeseman, 2018). After the topological analysis of personalized networks, additional genes were filtered as it follows (second WES filtering). Patient 1: *PTPN1*, *GRB2*, *ESR1*, *KIF4*, *MTFMT*, *MEOX2*, *TRAF2*, *EGFR*, *LLGL1*, *TNRC6A*, *HTT*, *NRP1*. Patient 2: *ESR1*, *MAPKAP1*, *LRRK2*, *RICTOR*, *RPTOR*, *LAMTOR1*, *LAMTOR5*, *MASP2*, *C4B_2*, *HTT*, *MYC*, *MLST8*, *CAV1*. Patient 3: *KMD1A*, *KMD3A*, *HPN*, *LRRK2*, *XRCC5*, *PARK7*, *ESR1*, *DAXX*, *DDX5*, *GRB2*, *ARID5A*, *FOXH1*, *PTPN1*, *CD33*, *PKN1*, *C1QC*, *SNW1*, *CALR*. Patient 4: *ESR1*, *LLGL1*, *HTT*, *KSR1*, *EWSR1*, *PIBF1*, *ATP2B4*.

### Criteria for Sequence Variants Selection in the Deductive Approach

All synonymous variants were excluded. In patient 1 (who inherited 16p13.11 microdeletion), and in patients 2 and 3 (with inherited 16p13.11p12.3 microdeletion) we selected hemizygous, recessive homozygous and compound heterozygous variants absent in healthy siblings carrying the 16p13.11 and 16p13.11p12.3 microdeletions, and heterozygous variants inherited from the non-carrier parent that were absent in both the healthy sibling and healthy parent carrier of the microdeletion. In patient 4, with *de novo* 16p13.11 microdeletion, all hemizygous, homozygous, compound heterozygous, and all heterozygous variants inherited from both healthy parents were selected. Variants showing a number of homozygotes higher than one in the general population were excluded (gnomAD v2.1.1 and v3.1), while all selected variants with a number of homozygotes equal to one or zero, or not annotated in the dataset, were included. The prediction of the effect on the protein structure and functionality of a single base variant was calculated using the PHRED quality score [CADD, Combined Annotation Dependent Depletion, genome build GRCh37 v1.4 (Rentzsch et al., 2019)]; the frameshift variants and the insertions or deletions involving two or more nucleotides were considered as deleterious. The hemizygous, homozygous, compound heterozygous or *de novo* heterozygous variants with PHRED <20.00 were considered, while the inherited heterozygous variants showing PHRED <20.00 were excluded.

The haploinsufficiency (HI) score (ClinGen Dosage Sensitivity Curation Page) and the gnomAD pLI value were considered for all genes showing variants selected as above. The haploinsufficiency of genes was expressed by HI score = 1 to 3, whereas the intolerance of the genes to variants causing loss of function (LoF) was expressed by gnomAD pLI value >0.9 (ClinGen Dosage Sensitivity Map). We have focused our attention on haploinsufficient genes or on genes showing high intolerance to variants causing LoF, since they might be more sensible to deleterious variants. All information about gene function and expression was taken from GeneCards and from GTEx Portal (Stelzer et al., 2016; The GTEx Consortium, 2020); the correlation of genes with Autosomal Recessive (AR)/Autosomal Dominant (AD)/X Linked disease was taken from OMIM database.

### Protein-Protein Interaction Networks

Cytoscape 3.8.2 was used to generate networks (Su et al., 2014). The public database IntAct was queried through Cytoscape using the PSICQUIC standard (the Proteomics Standard Initiative Common QUery InterfaCe). Identities of all genes showing at least one variant, as obtained by aCGH and WES, were used to generate a network encompassing all gene products and their first interactors. The network was filtered for human proteins to remove homology inferences. All self-loops and duplicated edges were removed. The topological analysis was performed using the NetworkAnalyzer tool in the Cytoscape environment. Nodes were prioritized by betweenness centrality as already reported (Zito et al., 2021). Most central nodes not included in the first WES filtering were checked for additional variants (second WES filtering; see Analyzed Genes). PHRED scores were annotated to nodes corresponding to altered genes. Arbitrary, out-of-range values were associated to deleted genes for color-coding purpose.

### Over-Representation Analysis

Over-representation analysis (ORA) was performed using WebGestalt (2019 release) by setting “geneontology_Biological_Process_noRedundant” as the enrichment database and “human genome, protein coding” as the reference database (Liao et al., 2019). *p*-values were calculated with the exact Fisher test and corrected for multiple testing according to the Benjamini-Hochberg method. The obtained GO (Gene Ontology) term with the lowest *p*-value was considered to check for the presence of variants in the enriched genes.

## Results

### Array CGH

Array CGH performed on DNA from four patients and their healthy parents and siblings showed a microdeletion in the short arm of chromosome 16, overlapping 16p13.11 region, in all patients, with different size. The characteristics of the microdeletions are described in Table 1. The microdeletion was inherited from an unaffected parent in Patient 1 (maternal inheritance), in Patient 2 (paternal inheritance) and in Patient 3 (maternal inheritance), while it was *de novo* in Patient 4. The protein-coding genes (according to OMIM, excluding RNA genes and pseudogenes) deleted in each patient are reported in Table 2. The genes *MPV17L*, *MARF1*, *NDE1*, *MYH11*, *CEP20*, *ABCC1*, *ABCC6* were present in an overlapping common region. No other CNVs classified as pathogenic, likely pathogenic or VOUS (variant of uncertain significance) were found in all four patients.

**TABLE 1:** Array CGH results.

Patient	Gender	CNV	Size (Mb)	Ref genome	Inheritance	Genomic coordinates
1	M	del16p13.11	1.35	GRCh37/hg19	Maternal	(15048732_16400833)
2	M	del16p13.11p12.3	3.03	GRCh37/hg19	Paternal	(15388706_18410892)
3	F	del16p13.11p12.3	3.03	GRCh37/hg19	Maternal	(15388706_18410892)
4	M	del16p13.11	1.34	GRCh37/hg19	*de novo*	(14968878_16311041)

**TABLE 2:** Genes encompassed by the 16p13.11 and 16p13.11p12.3 microdeletions in the four patients.

Patient 1	Patient 2	Patient 3	Patient 4
			*NOMO1*
*NPIPA1*			*NPIPA1*
*PDXDC1*			*PDXDC1*
*NTAN1*			*NTAN1*
*RRN3*			*RRN3*
*MPV17L*	*MPV17L*	*MPV17L*	*MPV17L*
*MARF1*	*MARF1*	*MARF1*	*MARF1*
*NDE1*	*NDE1*	*NDE1*	*NDE1*
*MYH11*	*MYH11*	*MYH11*	*MYH11*
*CEP20*	*CEP20*	*CEP20*	*CEP20*
*ABCC1*	*ABCC1*	*ABCC1*	*ABCC1*
*ABCC6*	*ABCC6*	*ABCC6*	*ABCC6*
*NOMO3*	*NOMO3*	*NOMO3*	
	*XYLT1*	*XYLT1*	

### Identification of Sequence Variants by Deductive Approach

**FIGURE 1:**
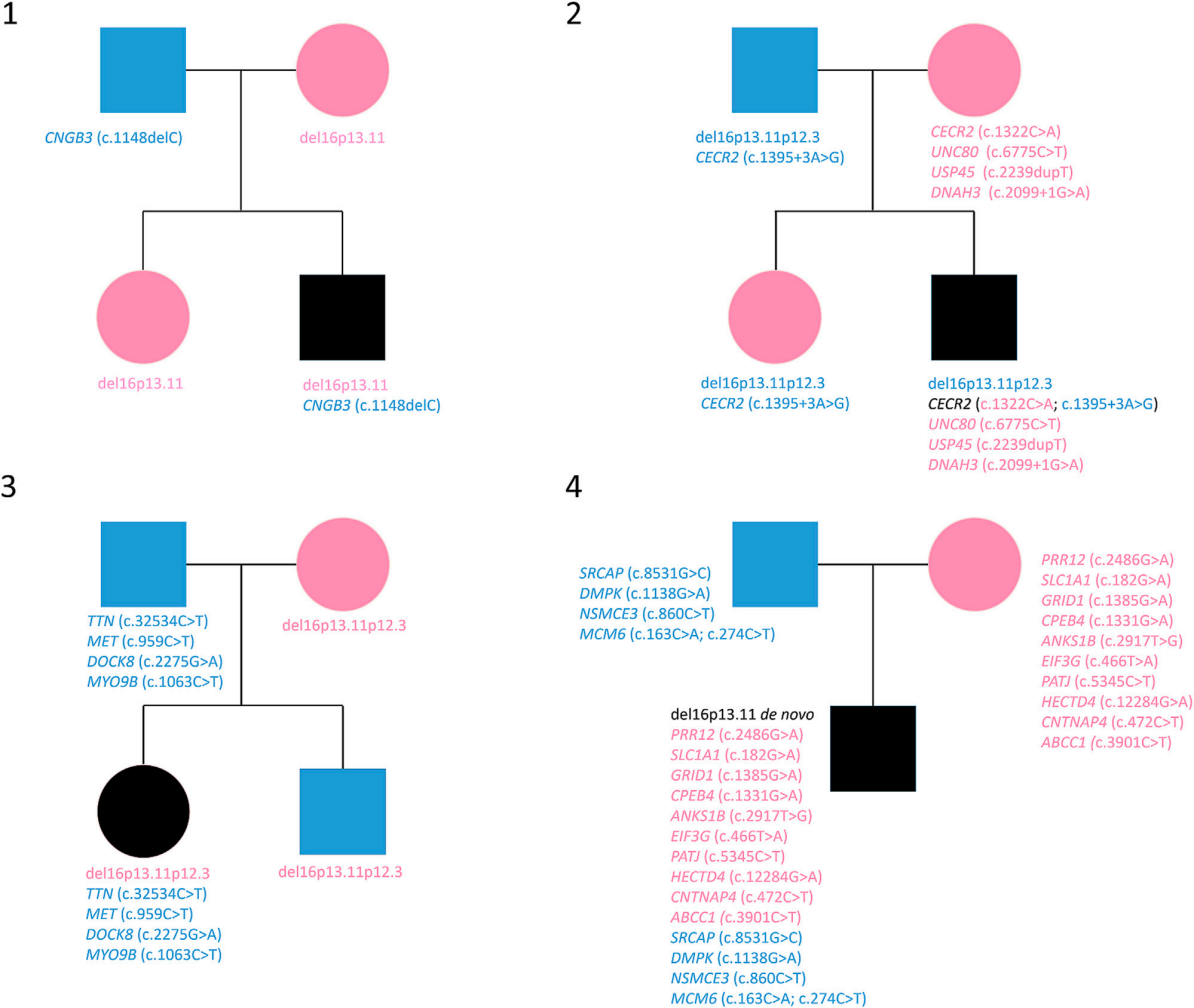
Variants reported in genealogical trees of the four families. Cyan: paternal inheritance. Magenta: maternal inheritance.

WES analysis was performed on four trios. The sequencing data are reported in Table 3 and visualized in genealogical trees (Figure 1)**.** In order to identify variants in genes included in the 16p13.11 deletion, as previously reported in some patients with microcephaly (Tan et al., 2017), all the deleted genes of each patient were sequenced. As a result, the heterozygous variant c.3901C>T in *ABCC1* was detected in Patient 4, whereas no variants were found in genes involved with NDE1 in cargo transport along the axon (see 2.4 Analyzed Genes). Patient 1 only showed the heterozygous paternal frame-shift variant c.1148delC in *CNGB3* gene. Patient 2 showed a compound heterozygosity in *CECR2* and three maternal heterozygous variants in *USP45*, *DNAH3* and *UNC80*. Concerning *CECR2*, the paternally inherited variant was c.1395+3A>G in an intron splice site region, whereas the maternal variant was the missense variant c.1322C>A. The maternally inherited heterozygous variants were the frame shift variant c.2239dupT in *USP45*, the intron splice site donor variant c.2099+1G>A in *DNAH3* and the missense variant c.6775C>T in *UNC80* gene. In Patient 3, paternal heterozygous missense variants c.32534C>T in *TTN*, c.959C>T in *MET*, c.2275G>A in *DOCK8* and c.1063C>T in *MYO9B* were detected. Patient 4 displayed paternal missense heterozygous variants in four genes: c.8531G>C in *SRCAP*, c.1138G>A in *DMPK*, c.860C>T in *NSMCE3*, c.274C>T and c.163C>A in *MCM6*. In another transcript, the variant c.1131+7G>A in an intron splice site region of *DMPK* was also observed. The maternal missense heterozygous variants are c.2486G>A in *PRR12*, c.182G>A in *SLC1A1*, c.1385G>A in *GRID1*, c.1331G>A in *CPEB4*, c.2917T>G in *ANKS1B*, c.466T>A in *EIF3G*, c.5345C>T in *PATJ*, c.12284G>A in *HECTD4*, c.472C>T in *CNTNAP4* and c.3901C>T in *ABCC1*, a gene encompassed by the 16p13.11 microdeletion.

**TABLE 3:** Variants selected with deductive approach from WES data in the four patients.

Patient	Sex	CNV	Inheritance	Gene symbol	HI score	pLI value	HGVSc	HGVSp	RefSeq	Max AF	Effect	PHRED score	Zygosity			
													Pat	Fat	Mot	Sib
1	M	del16p13.11	maternal	*CNGB3*	NA	0	c.1148delC	p.Thr383fs	NM_019098.4	3.4 × 10^−3^	FS	34.0	Het	Het		
2	M	del16p13.11p12.3	Paternal	*CECR2*	NA	1	c.1395+3A>G		NM_001290046.1	9.6 × 10^−6^	ISSR	14.7	Het	Het		Het
				*CECR2*			c.1322C>A	p.Ser441Tyr	NM_001290046.1	4.0 × 10^−6^	NSC	23.8	Het		Het	
				*USP45*	NA	0	c.2239dupT	p.Tyr747fs	NM_001080481.1	8.0 × 10^−6^	FS	33.0	Het		Het	
				*DNAH3*	NA	0	c.2099+1G>A		NM_017539.2	1.2 × 10^−4^	ISSD	28.9	Het		Het	
				*UNC80*	NA	0.05	c.6775C>T	p.His2259Tyr	NM_032504.1	3.2 × 10^−5^	NSC	21.9	Het		Het	
3	F	del16p13.11p12.3	maternal	*TTN*	NA	0	c.32534C>T	p.Thr10845Ile	NM_003319.4	3.6 × 10^−4^	NSC	23.2	Het	Het		
				*MET*	0	0.97	c.959C>T	p.Ala320Val	NM_000245.3	1.3 × 10^−3^	NSC	28.2	Het	Het		
				*DOCK8*	NA	0	c.2275G>A	p.Val759Met	NM_203447.3	2.6 × 10^−4^	NSC	24.5	Het	Het		
				*MYO9B*	NA	1	c.1063C>T	p.Leu355Phe	NM_004145.3	6.0 × 10^−4^	NSC	26.0	Het	Het		
4	M	del16p13.11	*de novo*	*SRCAP*	1	1	c.8531G>C	p.Gly2844Ala	NM_006662.2	1.3 × 10^−3^	NSC	23.0	Het	Het		
				*DMPK*	NA	0.04	c.1138G>A	p.Gly380Arg	NM_004409.4	2.0 × 10^−4^	NSC	28.7	Het	Het		
				*NSMCE3*	NA	0.06	c.860C>T	p.Ala287Val	NM_138704.3	1.2 × 10^−4^	NSC	22.3	Het	Het		
				*MCM6*	NA	0.98	c.274C>T	p.Arg92Trp	NM_005915.5	1.4 × 10^−3^	NSC	33.0	Het	Het		
				*MCM6*			c.163C>A	p.Arg55Ser	NM_005915.5	3.5 × 10^−4^	NSC	22.6	Het	Het		
				*PRR12*	NA	1	c.2486G>A	p.Arg829His	NM_020719.2	1.2 × 10^−3^	NSC	20.4	Het		Het	
				*SLC1A1*	NA	0	c.182G>A	p.Arg61Gln	NM_004170.5	2.3 × 10^−4^	NSC	29.7	Het		Het	
				*GRID1*	0	1	c.1385G>A	p.Arg462His	NM_017551.2	1.2 × 10^−4^	NSC	23.8	Het		Het	
				*CPEB4*	NA	0.99	c.1331G>A	p.Arg444His	NM_030627.3	2.3 × 10^−4^	NSC	22.5	Het		Het	
				*ANKS1B*	NA	1	c.2917T>G	p.Leu973Val	NM_152788.4	3.2 × 10^−5^	NSC	23.5	Het		Het	
				*EIF3G*	NA	0.99	c.466T>A	p.Ser156Thr	NM_003755.4	0	NSC	22.6	Het		Het	
				*PATJ*	NA	0	c.5345C>T	p.Ser1782Leu	NM_176877.2	2.4 × 10^−3^	NSC	23.9	Het		Het	
				*HECTD4*	NA	1	c.12284G>A	p.Arg4095Gln	NM_001109662.3	3.6 × 10^−4^	NSC	34.0	Het		Het	
				*CNTNAP4*	NA	0	c.472C>T	p.Pro158Ser	NM_033401.4	1.2 × 10^−5^	NSC	27.5	Het		Het	
				*ABCC1*	NA	0	c.3901C>T	p.Arg1301Cys	NM_004996.3	8.3 × 10^−4^	NSC	25.2	Het		Het	

HGVSc, Human Genome Variation Society coding sequence name; HGVSp, Human Genome Variation Society protein sequence name; FS, frame shift; ISSD, Intron, splice site donor; ISSR, Intron, splice site region; NSC, Non Synonimous Coding; RefSeq, NCBI nucleotide reference sequence ID; Max AF, maximum allele frequency; Pat, patient; Fat, father; Mot, mother; Sib, sibling; NA, not assigned; Het, heterozygous.

### Topological Analysis of Personalized Protein-Protein Interaction Networks

**FIGURE 2:**
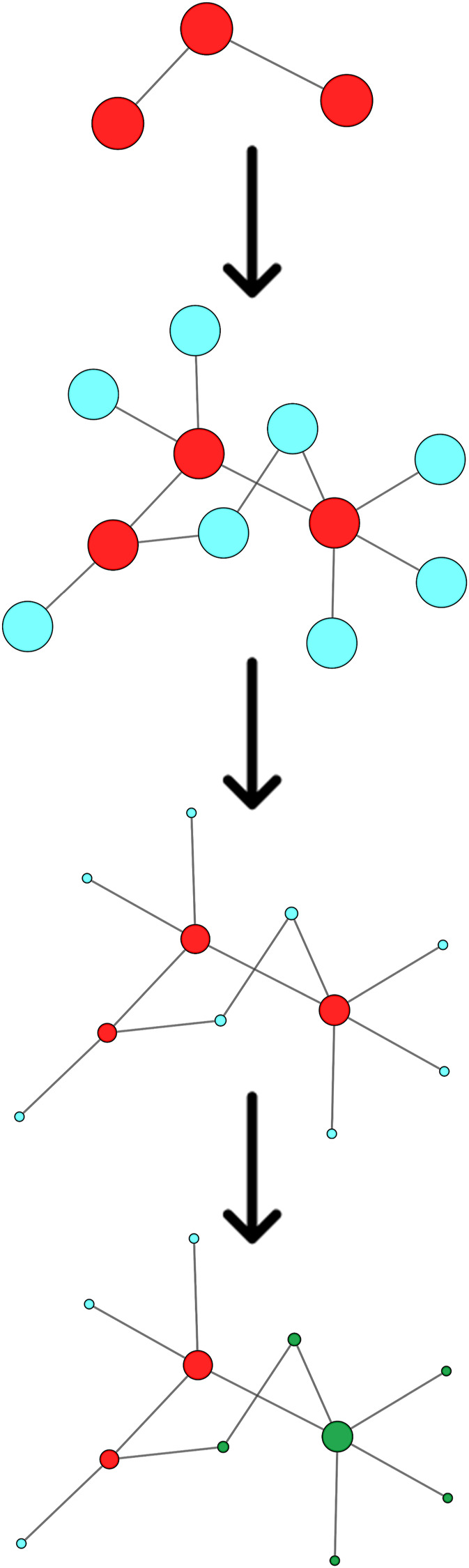
Schematic flowchart of the Systems biology approach. From top to bottom: A gene set (red nodes) is obtained for each patient. Interactors (blue nodes) are obtained from public databases. Centrality is calculated by topological analysis. Nodes overlapping with a GO gene set (green nodes) are identified.

**FIGURE 3:**
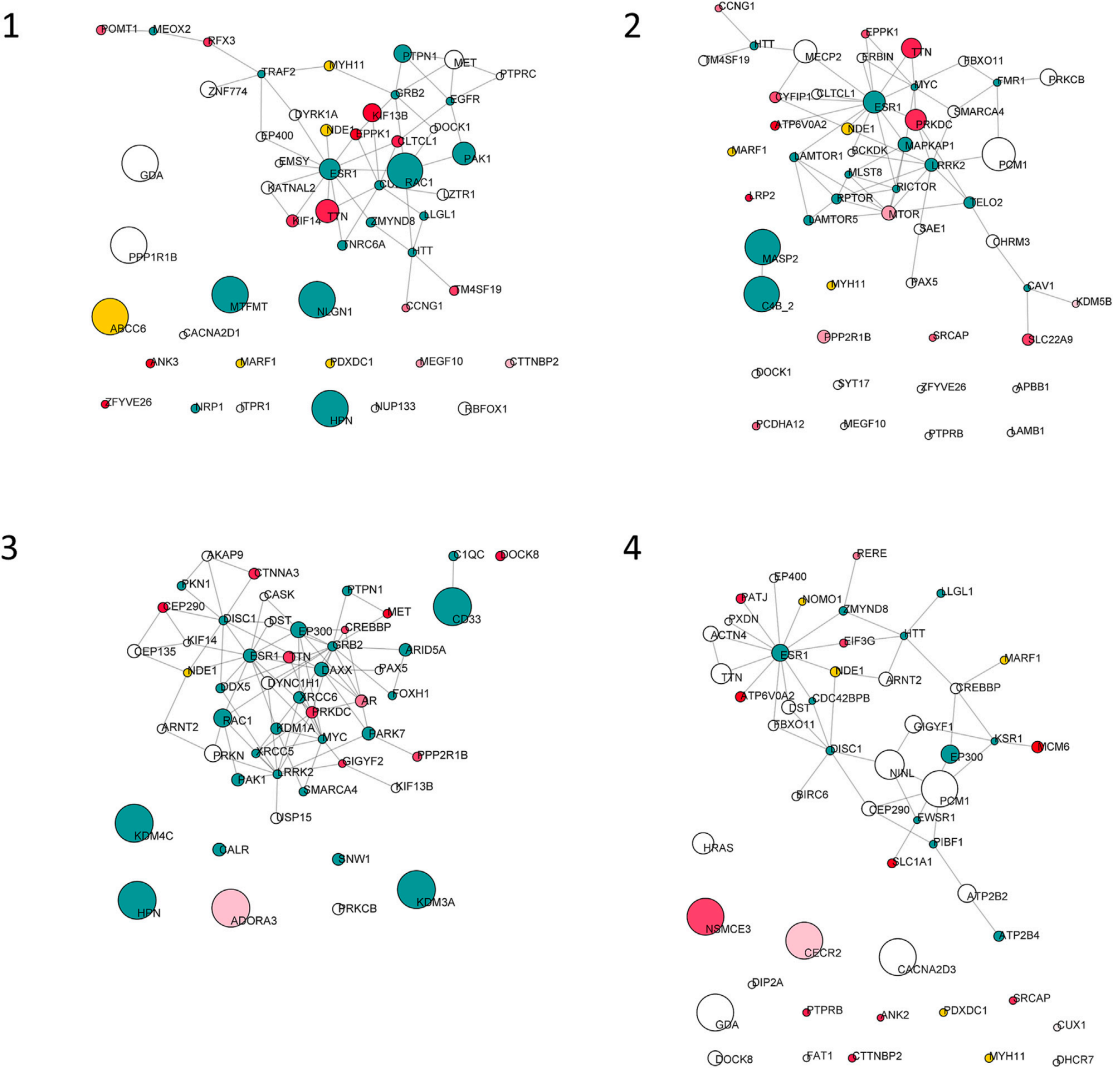
Top 50 nodes based on betweenness centrality. Node size is proportional to betweenness centrality. Node color is mapped on PHRED score as follows: white, score from 0 to 15; red shades, score from 15 to 20; red, score higher than 20; yellow, genes of the del16p13.11 microdeletion; green, unmapped.

**FIGURE 4:**
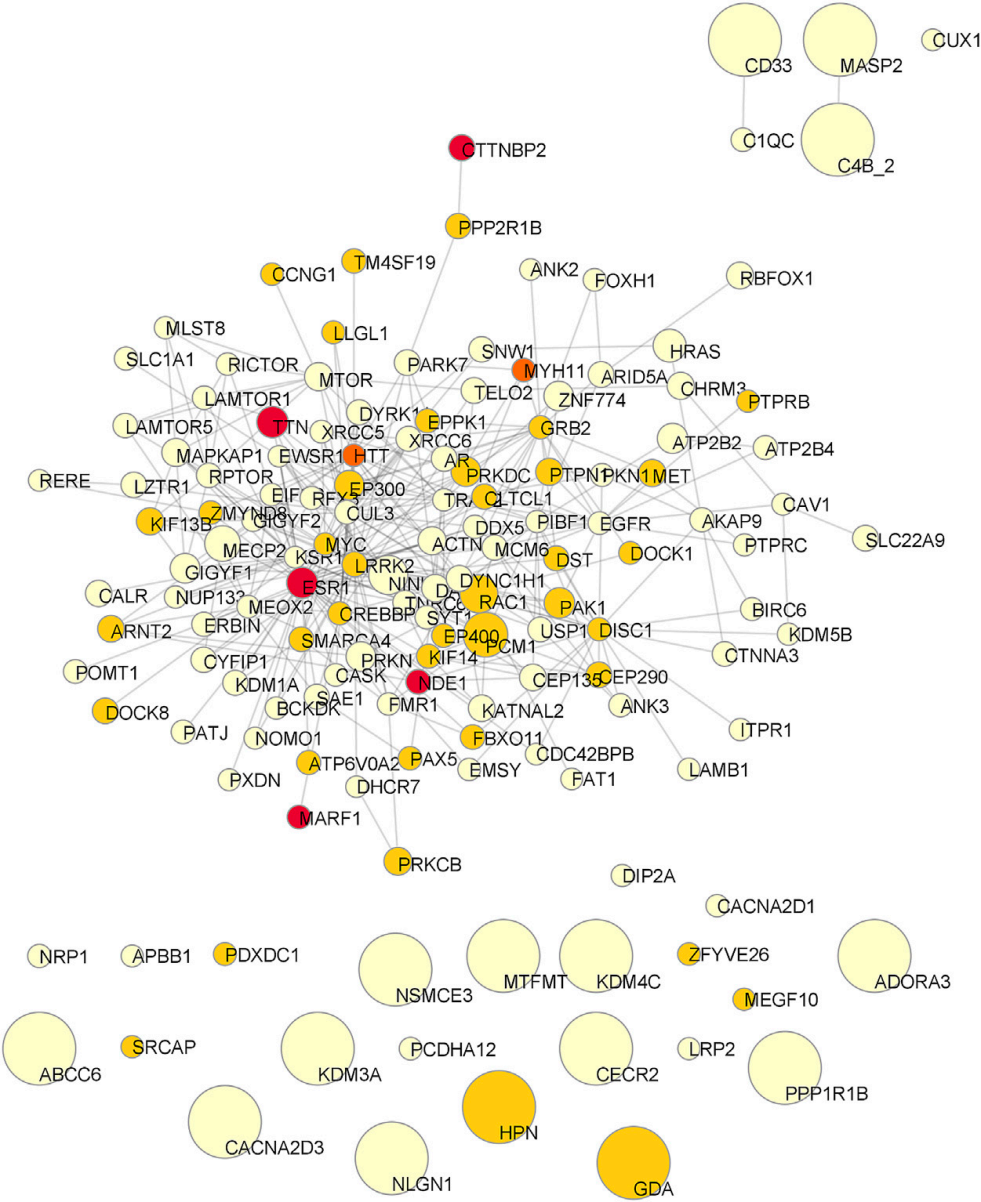
Consensus network of the Top50 genes of the four patients. Node size is proportional to the mean betweenness centrality in original networks. Color is associated to the number of observations (pale yellow: one patient; pale orange: two patients; dark orange: three patients; red: all patients).

Starting from the complete list of altered variants, a PPI network for each patient was built and analyzed to obtain betweenness centrality values for each node. Briefly, a gene signature is obtained for each patient that includes all variants without any a priori filtering. Then, edges are placed according to interaction evidence in public databases. Topological analysis is performed to quantify centrality parameters. Eventually, the network is compared to the GO database in order to find overlapping genes in the network and in the GO gene sets (Figure 2). Figure 3 shows subnetworks encompassing the 50 nodes with highest betweenness centrality for each patient. Genes that showed variants or belonged to the microdeletion region were identified with distinct colors (see caption). By merging the four lists of 50 genes each, a non-redundant list of 145 genes was obtained, with distinct centrality values for the same gene in distinct patients (Supplementary Table S1). Five nodes (i.e., *NDE1*, *MARF1*, *ESR1*, *CTTNBP2,* and *TTN*) were present in all four subnetworks, whereas *MYH11* and *HTT* were present in three out of four models (Figure 4). Several nodes showed high centrality, although they did not show variants or they showed variants filtered out after WES analysis (green nodes). Therefore, WES results were filtered again including the “large green” nodes, i.e., nodes that were not included in the prioritization step and that showed high betweenness centrality. According to this second filtering, new variants with potential pathogenicity were discovered (Table 4).

**TABLE 4:** New single nucleotide variants emerged from topological analysis of the four personalized networks.

PATIENT	Gene	HGVSC	Inheritance	PHRED score
1	*—*	*—*	*—*	*—*
2	*MASP2*	c.352C>T	Paternal	24.6
2	*RICTOR*	c.5084C>T	Paternal	13.6
3	*ARID5A*	c.932G>A	Paternal	13.0
3	*LRRK2*	c.5186C>T	Maternal	29.9
3	*LRRK2*	c.6241A>G	Paternal	24.1
4	*ESR1*	c.316A>G	Paternal	25.8
4	*ESR1*	c.*6623G>T	Paternal	0.5

HGVSc: Human Genome Variation Society coding sequence.

### Over-Representation Analysis of Personalized Protein-Protein Interaction Networks

The four subnetworks (Figure 3) were analyzed to identify functionally enriched gene ontologies (GOs). Table 5 reports, for each patient, GO terms whose enrichment is statistically significant in the over-representation analysis and the panel of genes that are present in both the subnetwork and in the geneset with the lowest *p*-value; in each panel the mutated genes and the genes included in the 16p13 microdeletion are highlighted. The analysis of the 50 nodes showing top betweenness centrality values in Patient 1 led to the identification of the following significantly enriched GOs: microtubule polymerization or depolymerization; actomyosin structure organization; phagocytosis; regulation of protein serine/threonine kinase activity; positive regulation of cell adhesion. Concerning Patient 2, the following GOs were highlighted: TOR signaling; peptidyl-serine modification; regulation of anatomical structure size; epithelial cell proliferation; covalent chromatin modification; process utilizing autophagic mechanism; muscle tissue development; protein polymerization; synaptic vesicle cycle. Patient 3 also displayed several significantly enriched GOs, i.e., intracellular receptor signaling pathway; intrinsic apoptotic signaling pathway; covalent chromatin modification; morphogenesis of a branching structure; interaction with symbiont; regulation of DNA-binding transcription factor activity; regulation of intracellular transport; regulation of chromosome organization. On the other hand, the analysis of the network of Patient 4 showed a single enriched pathway (establishment or maintenance of cell polarity). Concerning Patients 1 and 4, the most significantly enriched GOs comprised the *NDE1* gene, contained in the deleted region.

**TABLE 5:** Over-representation analysis of the Top50 genes for each patient.

*Patient 1*					
Gene set	Description	Size	Expect	Ratio	FDR
GO:0031109	microtubule polymerization or depolymerization	108	0.328	18.3	5.28 × 10^−4^
GO:0031032	actomyosin structure organization	184	0.559	12.5	5.28 × 10^−4^
GO:0006909	phagocytosis	238	0.723	9.68	1.94 × 10^−3^
GO:0071900	regulation of protein serine/threonine kinase activity	497	1.51	5.30	1.55 × 10^−2^
GO:0045785	positive regulation of cell adhesion	392	1.19	5.88	1.55 × 10^−2^
*Gene set: GO:0031109 microtubule polymerization or depolymerization*					

Gene Symbol	Gene name	Entrez gene ID
DYRK1A^*^	dual specificity tyrosine phosphorylation regulated kinase 1A	1859
KIF14^*^	kinesin family member 14	9928
MET^*^	MET proto-oncogene, receptor tyrosine kinase	4233
NDE1°	nudE neurodevelopment protein 1	54820
PAK1	p21 (RAC1) activated kinase 1	5058
RAC1	Rac family small GTPase 1	5879
***Patient 2***		

Gene set	Description	Size	Expect	Ratio	FDR
GO:0031929	TOR signaling	111	0.367	21.8	2.29 × 10^−6^
GO:0018209	peptidyl-serine modification	305	1.01	7.93	2.72 × 10^−3^
GO:0090066	regulation of anatomical structure size	487	1.61	5.58	5.73 × 10^−3^
GO:0050673	epithelial cell proliferation	372	1.23	6.41	5.73 × 10^−3^
GO:0016569	covalent chromatin modification	468	1.55	5.17	1.77 × 10^−2^
GO:0061919	process utilizing autophagic mechanism	473	1.56	5.11	1.77 × 10^−2^
GO:0060537	muscle tissue development	371	1.23	5.70	2.16 × 10^−2^
GO:0051258	protein polymerization	270	0.893	6.72	2.38 × 10^−2^
GO:0099504	synaptic vesicle cycle	193	0.638	7.83	3.28 × 10^−2^
*Gene set: GO:0031929 TOR signaling*					

Gene Symbol	Gene name	Entrez gene ID
LAMTOR1	late endosomal/lysosomal adaptor, MAPK and MTOR activator 1	55004
LAMTOR5	late endosomal/lysosomal adaptor, MAPK and MTOR activator 5	10542
MAPKAP1	mitogen-activated protein kinase associated protein 1	79109
MLST8	MTOR associated protein, LST8 homolog	64223
MTOR^*^	mechanistic target of rapamycin kinase	2475
RICTOR^*^	RPTOR independent companion of MTOR complex 2	253260
RPTOR	regulatory associated protein of MTOR complex 1	57521
TELO2	telomere maintenance 2	9894
***Patient 3***		

Gene set	Description	Size	Expect	Ratio	FDR
GO:0,030,522	intracellular receptor signaling pathway	284	0.959	12.5	1.06 × 10^−7^
GO:0097193	intrinsic apoptotic signaling pathway	285	0.962	9.35	8.51 × 10^−5^
GO:0016569	covalent chromatin modification	468	1.58	6.33	5.01 × 10^−4^
GO:0001763	morphogenesis of a branching structure	196	0.662	10.6	5.59 × 10^−4^
GO:0051702	interaction with symbiont	74	0.250	20.0	5.87 × 10^−4^
GO:0051090	regulation of DNA-binding transcription factor activity	404	1.364	6.60	7.57 × 10^−4^
GO:0032386	regulation of intracellular transport	422	1.42	6.32	9.55 × 10^−4^
GO:0033044	regulation of chromosome organization	329	1.11	7.20	1.10 × 10^−3^
*Gene set: GO:0030522 intracellular receptor signaling pathway*					

Gene Symbol	Gene name	Entrez gene ID
AR*	androgen receptor	367
CALR	calreticulin	811
DAXX	death domain associated protein	1616
DDX5	DEAD-box helicase 5	1655
EP300	E1A binding protein p300	2033
ESR1	estrogen receptor 1	2099
FOXH1	forkhead box H1	8928
KDM3A	lysine demethylase 3A	55818
PAK1	p21 (RAC1) activated kinase 1	5058
PARK7	Parkinsonism associated deglycase	11315
***Patient 4***		

Gene set	Description	Size	Expect	Ratio	FDR
GO:0007163	establishment or maintenance of cell polarity	203	0.620	11.3	2.04 × 10^−3^
*Gene set: GO:0007163 establishment or maintenance of cell polarity*					

Gene Symbol	Gene name	Entrez gene ID
CDC42BPB	CDC42 binding protein kinase beta	9578
DOCK8^*^	dedicator of cytokinesis 8	81704
DST^*^	dystonin	667
FAT1^*^	FAT atypical cadherin 1	2195
HTT	huntingtin	3064
LLGL1	LLGL scribble cell polarity complex component 1	3996
NDE1°	nudE neurodevelopment protein 1	54820

Ontology database: GO BP non-redundant. Sequence reference database: genome, human. Redundancy reduction: Weighted set cover. For the most significant GO gene set, genes overlapping with the Top50 gene set are shown. Size: number of genes in the GO Gene Set. Expect: expected number of overlapping genes. Ratio: ratio between actual and expected overlapping genes. FDR: Fisher test *p*-value after Benjamini-Hochberg correction; ^*^, Sequence variant; °, Deletion.

## Discussion

The 16p13.11 microdeletion has been reported in several cases in association with epilepsy, multiple congenital anomalies and cognitive impairment, but it has been also observed in normal subjects, so that a non-clear pathological significance can be attributed to this CNV (Paciorkowski et al., 2013; Tropeano et al., 2014). The variability of the phenotypic and clinical presentation of the 16p13.11 microdeletion in affected subjects may be due to incomplete penetrance, variable expressivity, unmasking of a recessive variant, different size of the microdeletion, variants in genes not involved in the deletion, or a combination of these conditions.

In order to identify the presence of a possible second hit mutational event or other pathological variants in genes outside the deletion, and their significance, we performed WES in four del16p13.11 patients and their unaffected relatives carrying the microdeletion. We did not find variants in genes included in the 16p deleted region in Patients 1, 2 and 3, so we excluded that their phenotype was due to the unmasking of a recessive variant. In Patient 4, with a *de novo* 16p13.11 microdeletion, we identified a maternal heterozygous variant in the *ABCC1* gene. ABCC1 (ATP binding cassette subfamily C member 1) is a molecular transporter with ATPase activity, not expressed in brain, mediating the export of organic anions and drugs from the cytoplasm and the exchange of various molecules across extra- and intra-cellular membranes. Moreover, it was shown to be involved in multi-drug resistance (Nasr et al., 2020). The *ABCC1* gene is related to autosomal dominant deafness-77 (DFNA77, OMIM #618915), a phenotype not observed in the patient and his mother, and did not show haploinsufficiency or LoF intolerance. For all these reasons, this variant does not seem to be an unmasked recessive variant with a pathological significance.

The selected variants obtained from WES highlighted a gene with compound heterozygosity in Patient 2 and heterozygous variants inherited from the parent without the 16p13.11 microdeletion in Patients 1, 2, 3. Additionally, heterozygous variants inherited from both parents were found in Patient 4. No variants were found in genes acting in axonal cargo transfer together with *NDE1*, the most important candidate gene for the neurodevelopmental phenotype encompassed by the 16p13.11 microdeletion, thus this function does not appear to be compromised.

Following a Systems medicine approach, the complete set of variants was used to build personalized models for each patient, in which altered genes are connected together either directly or by common interactors. To identify genes that potentially play a key role in the pathogenetic mechanism, each network model was analyzed to identify most central nodes (and, in turn, genes). This analysis generated several important findings. First, some of the genes in the microdeletion are often present in the first 50 positions (top50 subset), when nodes are ranked by betweenness centrality (Zito et al., 2021). Moreover, deleted genes may display interactions with mutated genes. In particular, *NDE1* was always observed in the Top50 subsets and interacted either directly or through common interactors with other mutated genes, suggesting its potential role in a common pathogenetic mechanism. The second aspect that emerged from the analysis of personalized network models was the presence of very central nodes that were not included in the first prioritization of variants. Indeed, we found that first interactors of altered genes that were observed to be highly central in networks were actually altered in their sequence. *ESR1*, which played a central role in all four models, appeared to be altered in Patient 4 only, with two paternally inherited single nucleotide variants (SNVs), one of them with a high PHRED score. ESR1 is known to mediate the effect of endocrine disruptors in impairing neurodevelopment (Xu et al., 2020). Another interesting finding was the identification for Patient 3 of two SNVs in compound heterozygosity in *LRRK2*, both showing high PHRED score. Actually, LRRK2 is a kinase with several activities and several variants were reported (OMIM *609007). The most common one is the G2019S (c.6055G>A), that is, responsible of an AD form of Parkinson’s disease (PARK8, OMIM #607060). Interestingly, variants of *LRRK2* were also associated to cognitive development leading to intellectual disability and ASD (Labonne et al., 2020). Hereafter, variants observed in each patient are discussed.

In Patient 1, a male patient with maternal 16p13.11 microdeletion, dysmorphisms and mild intellectual disability, the selected heterozygous paternal variant is a frame-shift variant in *CNGB3* (OMIM *605080), a gene encoding the beta subunit of a cyclic nucleotide-gated ion channel with a possible role in modulation of channel function in cone photoreceptors. This gene is not expressed in brain and is not related to AR or AD diseases, neither it shows haploinsufficiency or LoF intolerance. For all these reasons, although *CNGB3* is an emerging SFARI gene with score = 3, we exclude correlation of the present variant with the patient’s phenotype. No additional variants were identified after the second WES filtering. Concerning the functional enrichment analysis, we observed a significant over-representation of microtubule polymerization and depolymerization. Among the proteins responsible for this significance, we found NDE1. The corresponding gene is included within the microdeletion region and the encoded protein is a member of the nuclear distribution E (NudE) family, with a role in microtubule organization and neurodevelopment (Monda and Cheeseman, 2018). Although *NDE1* has been associated to microhydranencephaly and lissencephaly-4 (OMIM #605013 and #614019, respectively), our observation supports its role in neurodevelopmental disorders in the absence of cerebral malformations (Allach El Khattabi et al., 2020). Variants were found in other genes of the enriched ontology, *i.e.*, *DYRK1A* (PHRED = 9.75), *KIF14* (PHRED = 23.00) and *MET* (PHRED = 6.39). Therefore, we may hypothesize that the microtubule polymerization and depolymerization pathway is impaired in this patient and could be at the origin of the observed clinical phenotype. The role of 16p13.11 microdeletion in determining the complex phenotype of Patient 1 can be suggested by the involvement of *NDE1* in microtubule functionality pathways and the CNV may be considered as a contributing cause variant.

Patient 2, a male patient with a paternal 16p13.11p12.3 microdeletion and with severe neuropsychiatric phenotype, showed a compound heterozygosity in *CECR2* (Histone Acetyl-Lysine Reader; OMIM *607576) gene, composed by a paternal variant in an intron splice site region and by a maternal missense variant. This genetic condition is unique to the patient and is not present in other healthy members of the family with or without 16p13.11 microdeletion. *CECR2*, highly expressed in cerebellum, is involved in chromatin remodeling and may play a role in DNA damage response and in several developmental processes. CECR2 is a component of the CECR2-containing remodeling factor (CERF), involved in neurulation and postnatal brain development (Banting et al., 2005). No AD or AR diseases are known to be related to *CECR2* in OMIM*.* The observed compound heterozygosity in *CECR2* seems to be a possible cause of the patient’s phenotype. The maternal heterozygosities were a frame-shift variant in *USP45* (OMIM *618439), an intron splice site donor region variant in *DNAH3* (OMIM *603334) and a missense variant in *UNC80* (OMIM *612636). None of these three genes were related to AD diseases or showed haploinsufficiency or LoF intolerance, nor they were among the top50 genes in the network model of Patient 2. By performing ORA, we found that TOR signaling was the most significant and most enriched GO term, with eight geneset nodes overlapping with the network model (see Table 5). Among these eight nodes, we found variants in *MTOR* (c.2805G>A, PHRED = 18.86) and *RICTOR* (c.5084C>T, PHRED = 13.60) genes. These findings support the role of autophagy in neurodevelopmental diseases (Marsh and Dragich, 2019; Lv et al., 2020) with a specific reference to mTORopathies (Karalis and Bateup, 2021) that lead to epilepsy, ASD and, present in this patient, intellectual disability, obesity and hyperphagia. Worthy of note, *NDE1* was included in the top50 genes ranked by betweenness centrality, highlighting its role in the network topology. Therefore, we cannot exclude a possible pathogenic contribution of the microdeletion. On the other hand, it was not possible to significantly define its involvement in any biological pathway as described by GO.

Patient 3, a female patient with maternal 16p13.11p12.3 microdeletion with a mild neurocognitive phenotype, showed paternal missense heterozygous variants in *TTN* (OMIM *188840), *MET* (OMIM *164860), *DOCK8* (OMIM *611432) and *MYO9B* (OMIM *602129). The genes showing heterozygous variants inherited from the healthy father and related to AD diseases are *TTN*, related to AD myopathies and cardiomyopathies and *MET*, related to AD susceptibility to Osteofibrous Dysplasia (OMIM #607278). No signs of these pathologies are present in both Patient 3 and his father. *MYO9B* is a susceptibility factor to Celiac disease (OMIM #609753) and showed gnomAD pLI = 1. The protein encoded by *MET* is a hepatocyte growth factor receptor (HGFR); during embryonic development MET plays a role in neuronal precursors; in adults participates in organ regeneration and tissue remodeling and promotes differentiation and proliferation of hematopoietic cells. There is no evidence of a hypothetically causative role of these variants in determining the patient’s neurocognitive phenotype. The second filtering of WES data allowed us to identify two compound heterozygous SNVs in the *LRRK2* gene (c.1586C>T, PHRED = 29.9; c.6241A>G, PHRED = 24.1) and a paternal SNV in the *ARID5A* gene (c.932G>A, PHRED = 13.0). The over-representation analysis of the network model highlighted intracellular receptor signaling as the most significantly enriched pathway. Among the genes mapped on this GO term, *AR* showed two variants with different deleteriousness (c.636G>A, PHRED = 14.8, and c.1174C>T, PHRED = 20.5). As for Patient 2, *NDE1* was included in the Top50 genes, highlighting its role in the network topology. Again, we cannot exclude a possible pathogenic contribution of the microdeletion. On the other hand, it was not possible to significantly define its involvement in any biological pathway as described by GO.

In Patient 4, a male patient with a *de novo* 16p13.11 microdeletion diagnosed with non-syndromic ASD, we pointed our attention to mutated genes showing dosage sensitivity or LoF intolerance. The patient showed paternal variants in the haploinsufficient gene *SRCAP*, the core catalytic component of the chromatin remodeling complex. Among LoF intolerant genes, we found *MCM6* (OMIM *601806) with paternal heterozygosity and *PRR12* (OMIM *616633), *GRID1* (OMIM *610659), *CPEB4* (OMIM *610607), *ANKS1B* (OMIM *607815), *EIF3G* (OMIM *603813) and *HECTD4* (not found in OMIM) with maternal heterozygosity. *SRCAP,* with a missense variant, encodes the core catalytic component of the chromatin-remodeling SRCAP complex. The gene is related to AD Floating-Harbor syndrome (OMIM #136140) (Hood et al., 2012). *MCM6*, essential for the initiation of eukaryotic genome replication, is related to AD Lactase persistence/non-persistence (OMIM #223100) (Almazar et al., 2019) and has low expression in brain. *PRR12* encodes a proline-rich protein nuclear factor associated to neurodevelopmental disorders and intellectual disability (Leduc et al., 2018). No AD diseases are related to this gene. *GRID1* encodes a subunit of glutamate receptor channels mediating most of the fast excitatory synaptic transmission in the central nervous system and plays key roles in synaptic plasticity. No AD diseases are related to this gene. *CPEB4* has low expression in brain and plays a role in promoting tumor growth and progression. No AD diseases are related to this gene. We cannot exclude a hypothetical damaging role of this gene, known to be associated with autism (SFARI score = 2), in some function of central nervous system. *ANKS1B* is expressed in all cerebral districts, with a possible role in normal brain development. No AD diseases are related to this gene. *EIF3G* shows low expression in brain and is required for initiation of protein translation. No AD diseases are related to this gene*. HECTD4* encodes HECT Domain E3 ubiquitin-protein ligase; the gene is highly expressed at cerebellar level and is not related to AD diseases. No evidences of cited AD diseases are present in the patient and his parents. The second WES filtering highlighted two paternal SNVs in the *ESR1* gene, one of them with a high PHRED score (c.316A>G, PHRED = 25.8). The over-representation analysis of the Top50 network model suggested a single enriched GO, i.e., establishment or maintenance of cell polarity. Among enriched genes, we found the microdeletion gene *NDE1*, together with genes showing variants with low PHRED scores (*DOCK8*, *DST*, *FAT1*).

Our results suggest that performing WES in presence of 16p13.11 microdeletion is necessary and that the present dual deductive and inductive approach, when applied to other families, will help to clarify the clinical contribution of 16p13.11 microdeletion, when the non-deleted alleles of the genes included in the microdeletion do not show variants.

## Conclusion

In conclusion, we have analyzed WES data from four patients carrying the 16p13.11 microdeletion by both a deductive approach and a Systems medicine analysis based on network models. As a whole, we identified several variants potentially involved in the pathogenetic mechanism at the basis of their phenotype. In particular, no additional CNVs were found in the four patients. Accordingly, the low penetrance of the microdeletion and the variability of phenotypes cannot be explained by the presence of other CNVs. From WES analysis, no potential pathogenic variants were found in the non-deleted alleles of genes in 16p13.11 or 16p13.11p12.3 regions, thus excluding unmasked recessive conditions.

Concerning variants identified by WES, we did not identify relevant variants in Patient 1, with a mild phenotype. However, the microdeletion gene *NDE1* was found to be one of the enriched genes in the microtubule polymerization or depolymerization pathway, together with *DYRK1A*, *KIF14,* and *MET*, potentially explaining the observed phenotype. We identified a compound heterozygosity in *CECR2* in Patient 2, with a severe phenotype. Additionally, two SNVs were identified in *MTOR* and *RICTOR* genes, both present in the enriched TOR signaling ontology and central in the network model of Patient 2. We could therefore hypothesize a possible association among the clinical phenotype observed in Patient 2, its genotype and an mTORopathy. Patient 3, with a mild phenotype, showed heterozygous paternal variants in *TTN*, *MET*, *DOCK8* (all three central genes in the network model of this patient) and *MYO9B*. Moreover, the network topological analysis led to the identification of compound heterozygous SNVs in the *LRRK2* gene. Eventually, paternal heterozygous variants in *SRCAP* and *MCM6* and maternal heterozygous variants in *PRR12*, *GRID1*, *CPEB4*, *ANKS1B*, *EIF3G,* and *HECTD4* were found in Patient 4, diagnosed with non-syndromic ASD. Furthermore, *ESR1*, the most central gene in all network models, showed a SNV only in Patient 4, and the single significant GO included the microdeletion gene *NDE1*.

Concerning the role of 16p13.11 microdeletion in determining the neurodevelopmental phenotype of the four patients, we can conclude that the CNV can be considered a contributing cause variant in patients 1 and 4, since NDE1 is present in both Top50 networks and GO terms. Conversely, it may be considered a candidate factor in patients 2 and 3, since NDE1 is present with high centrality only in the Top50 networks, without being associated to the most significant enriched pathway.

Altogether, we have demonstrated that WES is a fundamental tool in the genetic investigation of patients having a predisposing variant, but this is not sufficient to define the clinical phenotype. It would be interesting to consider additional sequence variants in the gene set recently prioritized by Leblond et al. in the first filtering step (Leblond et al., 2021). Additionally, the analysis of WES data using Systems medicine tools, such as personalized network models, allowed us to prioritize genes on a high throughput scale and to discover variants in genes that were not prioritized at first. Indeed, the dual study approach, classical deductive on WES results and inductive by personalized network models, is a good strategy to clarify, in each patient, the clinical contribution of an uncertain or predisposing CNV. Additionally, uncovering novel enriched pathways associated to a specific phenotype may also provide new insights for personalized therapeutic strategies.

## Data Availability

The datasets presented in this study can be found in online repositories. The names of the repository/repositories and accession number(s) can be found below: https://www.ebi.ac.uk/eva/, European Variation Archive Project: PRJEB41629, Analyses: ERZ4208684 https://decipher.sanger.ac.uk/, 350680 (Patient 1), 414066 (Patient 2), 414067 (Patient 3), and 318359 (Patient 4).
