# Supplementary material for: Whole Exome Sequencing in 16p13.11 Microdeletion Patients Reveals New Variants Through Deductive and Systems Medicine Approaches
Source: Front Genet. 2022 Mar 15;13:798607. doi: 10.3389/fgene.2022.798607 (PMC8965081; doi:10.3389/fgene.2022.798607)
Supplement: Supplementary file 1 [file Table1.DOCX]

Supplementary Material

**Supplementary Table 1.** Betweenness centrality measures of Top50 nodes in each patient.

| NODE | Patient 1 | Patient 2 | Patient 3 | Patient 4 |
| --- | --- | --- | --- | --- |
| *ABCC6* | 1.000 | -- | -- | -- |
| *ACTN4* | -- | -- | -- | 0.073 |
| *ADORA3* | -- | -- | 1.000 | -- |
| *AKAP9* | -- | -- | 0.039 | -- |
| *ANK2* | -- | -- | -- | 0.016 |
| *ANK3* | 0.031 | -- | -- | -- |
| *APBB1* | -- | 0.019 | -- | -- |
| *AR* | -- | -- | 0.053 | -- |
| *ARID5A* | -- | -- | 0.053 | -- |
| *ARNT2* | -- | -- | 0.053 | 0.065 |
| *ATP2B2* | -- | -- | -- | 0.093 |
| *ATP2B4* | -- | -- | -- | 0.040 |
| *ATP6V0A2* | -- | 0.035 | -- | 0.039 |
| *BCKDK* | -- | 0.022 | -- | -- |
| *BIRC6* | -- | -- | -- | 0.028 |
| *C1QC* | -- | -- | 0.033 | -- |
| *C4B_2* | -- | 0.786 | -- | -- |
| *CACNA2D1* | 0.022 | -- | -- | -- |
| *CACNA2D3* | -- | -- | -- | 1.000 |
| *CALR* | -- | -- | 0.057 | -- |
| *CASK* | -- | -- | 0.026 | -- |
| *CAV1* | -- | 0.020 | -- | -- |
| *CCNG1* | 0.024 | 0.021 | -- | -- |
| *CD33* | -- | -- | 0.767 | -- |
| *CDC42BPB* | -- | -- | -- | 0.017 |
| *CECR2* | -- | -- | -- | 1.000 |
| *CEP135* | -- | -- | 0.061 | -- |
| *CEP290* | -- | -- | 0.038 | 0.068 |
| *CHRM3* | -- | 0.065 | -- | -- |
| *CLTCL1* | 0.051 | 0.043 | -- | -- |
| *CREBBP* | -- | -- | 0.018 | 0.042 |
| *CTNNA3* | -- | -- | 0.043 | -- |
| *CTTNBP2* | 0.030 | 0.116 | 0.049 | 0.023 |
| *CUL3* | 0.036 | -- | -- | -- |
| *CUX1* | -- | -- | -- | 0.018 |
| *CYFIP1* | -- | 0.049 | -- | -- |
| *DAXX* | -- | -- | 0.066 | -- |
| *DDX5* | -- | -- | 0.032 | -- |
| *DHCR7* | -- | -- | -- | 0.016 |
| *DIP2A* | -- | -- | -- | 0.021 |
| *DISC1* | -- | -- | 0.030 | 0.028 |
| *DOCK1* | 0.023 | 0.029 | -- | -- |
| *DOCK8* | -- | -- | 0.034 | 0.070 |
| *DST* | -- | -- | 0.026 | 0.065 |
| *DYNC1H1* | -- | -- | 0.056 | -- |
| *DYRK1A* | 0.048 | -- | -- | -- |
| *EGFR* | 0.027 | -- | -- | -- |
| *EIF3G* | -- | -- | -- | 0.025 |
| *EMSY* | 0.024 | -- | -- | -- |
| *EP300* | -- | -- | 0.078 | 0.094 |
| *EP400* | 0.038 | -- | -- | 0.028 |
| *EPPK1* | 0.048 | 0.028 | -- | -- |
| *ERBIN* | -- | 0.038 | -- | -- |
| *ESR1* | 0.119 | 0.132 | 0.061 | 0.085 |
| *EWSR1* | -- | -- | -- | 0.017 |
| *FAT1* | -- | -- | -- | 0.017 |
| *FBXO11* | -- | 0.045 | -- | 0.040 |
| *FMR1* | -- | 0.024 | -- | -- |
| *FOXH1* | -- | -- | 0.026 | -- |
| *GDA* | 1.000 | -- | -- | 1.000 |
| *GIGYF1* | -- | -- | -- | 0.087 |
| *GIGYF2* | -- | -- | 0.022 | -- |
| *GRB2* | 0.029 | -- | 0.034 | -- |
| *HPN* | 1.000 | -- | 1.000 | -- |
| *HRAS* | -- | -- | -- | 0.115 |
| *HTT* | 0.029 | 0.029 | -- | 0.021 |
| *ITPR1* | 0.023 | -- | -- | -- |
| *KATNAL2* | 0.060 | -- | -- | -- |
| *KDM1A* | -- | -- | 0.047 | -- |
| *KDM3A* | -- | -- | 1.000 | -- |
| *KDM4C* | -- | -- | 1.000 | -- |
| *KDM5B* | -- | 0.021 | -- | -- |
| *KIF13B* | 0.100 | -- | 0.032 | -- |
| *KIF14* | 0.056 | -- | 0.018 | -- |
| *KSR1* | -- | -- | -- | 0.018 |
| *LAMB1* | -- | 0.023 | -- | -- |
| *LAMTOR1* | -- | 0.034 | -- | -- |
| *LAMTOR5* | -- | 0.042 | -- | -- |
| *LLGL1* | 0.030 | -- | -- | 0.025 |
| *LRP2* | -- | 0.021 | -- | -- |
| *LRRK2* | -- | 0.065 | 0.029 | -- |
| *LZTR1* | 0.051 | -- | -- | -- |
| *MAPKAP1* | -- | 0.075 | -- | -- |
| *MARF1* | 0.030 | 0.029 | 0.053 | 0.025 |
| *MASP2* | -- | 0.464 | -- | -- |
| *MCM6* | -- | -- | -- | 0.048 |
| *MECP2* | -- | 0.139 | -- | -- |
| *MEGF10* | 0.023 | 0.019 | -- | -- |
| *MEOX2* | 0.025 | -- | -- | -- |
| *MET* | 0.102 | -- | 0.028 | -- |
| *MLST8* | -- | 0.022 | -- | -- |
| *MTFMT* | 1.000 | -- | -- | -- |
| *MTOR* | -- | 0.074 | -- | -- |
| *MYC* | -- | 0.019 | 0.027 | -- |
| *MYH11* | 0.040 | 0.031 | -- | 0.027 |
| *NDE1* | 0.052 | 0.055 | 0.024 | 0.038 |
| *NINL* | -- | -- | -- | 0.168 |
| *NLGN1* | 1.000 | -- | -- | -- |
| *NOMO1* | -- | -- | -- | 0.020 |
| *NRP1* | 0.031 | -- | -- | -- |
| *NSMCE3* | -- | -- | -- | 1.000 |
| *NUP133* | 0.023 | -- | -- | -- |
| *PAK1* | 0.133 | -- | 0.055 | -- |
| *PARK7* | -- | -- | 0.053 | -- |
| *PATJ* | -- | -- | -- | 0.034 |
| *PAX5* | -- | 0.041 | 0.023 | -- |
| *PCDHA12* | -- | 0.025 | -- | -- |
| *PCM1* | -- | 0.213 | -- | 0.216 |
| *PDXDC1* | 0.032 | -- | -- | 0.023 |
| *PIBF1* | -- | -- | -- | 0.023 |
| *PKN1* | -- | -- | 0.029 | -- |
| *POMT1* | 0.035 | -- | -- | -- |
| *PPP1R1B* | 1.000 | -- | -- | -- |
| *PPP2R1B* | -- | 0.063 | 0.032 | -- |
| *PRKCB* | -- | 0.094 | 0.048 | -- |
| *PRKDC* | -- | 0.125 | 0.049 | -- |
| *PRKN* | -- | -- | 0.086 | -- |
| *PTPN1* | 0.095 | -- | 0.035 | -- |
| *PTPRB* | -- | 0.022 | -- | 0.021 |
| *PTPRC* | 0.022 | -- | -- | -- |
| *PXDN* | -- | -- | -- | 0.019 |
| *RAC1* | 0.223 | -- | 0.093 | -- |
| *RBFOX1* | 0.062 | -- | -- | -- |
| *RERE* | -- | -- | -- | 0.018 |
| *RFX3* | 0.031 | -- | -- | -- |
| *RICTOR* | -- | 0.025 | -- | -- |
| *RPTOR* | -- | 0.043 | -- | -- |
| *SAE1* | -- | 0.045 | -- | -- |
| *SLC1A1* | -- | -- | -- | 0.031 |
| *SLC22A9* | -- | 0.053 | -- | -- |
| *SMARCA4* | -- | 0.057 | 0.027 | -- |
| *SNW1* | -- | -- | 0.050 | -- |
| *SRCAP* | -- | 0.022 | -- | 0.021 |
| *SYT17* | -- | 0.034 | -- | -- |
| *TELO2* | -- | 0.054 | -- | -- |
| *TM4SF19* | 0.036 | 0.045 | -- | -- |
| *TNRC6A* | 0.039 | -- | -- | -- |
| *TRAF2* | 0.023 | -- | -- | -- |
| *TTN* | 0.135 | 0.116 | 0.049 | 0.106 |
| *USP15* | -- | -- | 0.044 | -- |
| *XRCC5* | -- | -- | 0.027 | -- |
| *XRCC6* | -- | -- | 0.050 | -- |
| *ZFYVE26* | 0.025 | 0.024 | -- | -- |
| *ZMYND8* | 0.037 | -- | -- | 0.028 |
| *ZNF774* | 0.086 | -- | -- | -- |

**Notes:** Node names correspond to gene symbols. 145 gene IDs in total.
